# Exploring the link between nitrate exposure and thyroid cancer: A nationwide state-level analysis

**DOI:** 10.1210/jendso/bvag019

**Published:** 2026-01-27

**Authors:** Christina Zanazanian, Jason Semprini

**Affiliations:** Department of Public Health, Des Moines University, West Des Moines, IA 50266, USA; Department of Public Health, Des Moines University, West Des Moines, IA 50266, USA

**Keywords:** cancer, thyroid, prevention, environment, early-onset, epidemiology

## Abstract

**Context:**

Early-onset thyroid cancer incidence has been increasing, raising concerns about contributing factors.

**Objective:**

We aimed to investigate the role of nitrate contamination in drinking water as a contributor to early-onset thyroid cancer incidence.

**Methods:**

We designed an ecological study, analyzing population-based data from the National Program of Cancer Registries. We quantified the association between nitrate exposure and early-onset thyroid cancer with a set of Poisson generalized estimating equations (GEE). Analyses adjusted for current and 7-year lagged obesity rates and access to screening. Our nationwide study includes data from all 50 states in the United States. Patients between ages 0 to 14, 15 to 39, and 40 to 59 who were diagnosed with thyroid cancer between 2003 and 2022. States were categorized into 2 groups based on predicted groundwater nitrate levels. States with nitrate < 2.0 mg/L were classified as “Low,” and states with levels ≥ 2.0 mg/L as “High.” Population-adjusted cases of thyroid cancer incidence, with patients grouped by age and sex.

**Results:**

For the 0 to 14 or 40 to 59 age groups, we found no differences in thyroid cancer incidence by nitrate exposure. For ages 15 to 39, there were 41.6 (CI: 6.2, 77.1) more cases in high nitrate states, reflecting an 18.5% difference. Stratified by sex, in the 15 to 39 age group of females had 32.7 (CI: 6.3, 59.1) more cases and males had 8.2 (CI: 0.1, 16.4) more cases, reflecting a 17.8% and 20.4% difference respectively.

**Conclusion:**

Elevated exposure to groundwater nitrate may be a significant preventable contributor to thyroid cancer in adolescents and young adults.

Thyroid cancer is one of the most common cancers diagnosed in young people under age 60 [1]. Early-onset thyroid cancer incidence has increased for decades, with exceptional growth in pediatric/adolescent young adult (AYA) populations [2, 3]. While family history, genetic mutations, and obesity are the most common drivers of thyroid cancer incidence, environmental exposures, such as radiation, also play a role [4-6]. Although underexplored, there is also a strong biological mechanistic basis for nitrate's role in thyroid cancer etiology [7].

Nitrate, a naturally occurring compound, is increasingly found in agricultural fertilizers, and often runs off of crop fields contaminating public and private drinking water [8]. Once consumed, nitrate acts as a competitive inhibitor of the sodium iodide symporter and prevents iodide uptake by the thyroid gland, impairing thyroid hormone synthesis [9-11]. This impairment leads to elevated levels of thyroid stimulating hormone, which can result in hypertrophy, thyroid disease, hyperplasia, and potentially malignant tumors [12-15]. Beyond hormonal impairment, nitrate consumption further contributes to carcinogenesis risk through the reduction of nitrate to nitrite, which facilitates the formation of N-nitroso compounds which have shown to increase tumor risk in vivo [15].

Despite the multiple potential mechanisms supporting the biological plausibility linking nitrate exposure to thyroid cancer, existing epidemiological evidence has been limited. Among the first epidemiological evaluations, a study in the agricultural state of Iowa revealed that longer exposure to higher levels of nitrate was associated with increased risk of thyroid cancer [7]. More recent evidence from California further supports the potential association between nitrate and thyroid cancer risk [16]. Notably, in both studies, the level of exposure was only half the “safe” regulatory threshold [17]. Whether the results from these 2 studies generalize beyond Iowa and California, and to younger age groups, remains unclear.

As nitrate levels continue to rise in our nation's groundwater, public health systems must respond to the drinking water contamination and possible health effects of chronic, elevated nitrate consumption [18-20]. Given the dearth of studies on thyroid cancer, especially in younger populations, and the expected toll of nitrate contamination on the burden of thyroid cancer [21], our study aimed to conduct a novel nationwide, population-based analysis quantifying the association between nitrate exposure and early-onset thyroid cancer.

## Methods

### Data and measures

Our outcome was a state-level, annual measure of thyroid cancer incidence. Cancer incidence data were obtained from the National Program of Cancer Registries (NPCR) via the Surveillance, Epidemiology, and End Results (SEER)*Stat statistical software [22]. The NPCR provides population-based cancer incidence data covering the entire U.S. population. All data was aggregated at the state-level and included all 50 states (plus D.C.). We restricted case selection to individuals diagnosed with thyroid cancer between 2003 and 2022, ensuring 20 years of complete, non-missing data. To focus on early-onset cancer, we also restricted incidence to ages 0-59. Incidence rates were further stratified by sex and by age group, categorized as pediatric (0-14 years), adolescent and young adult (AYA; 15-39 years), and early-onset adult (40-59 years).

Our exposure of interest was a binary measure classifying states as above/below median groundwater nitrate levels. Accessing county-level groundwater nitrate data from the United States Geological Survey (USGS) [23] (1991-2010), we first estimated a population weighted average of each state's groundwater nitrate level. States with average nitrate levels < 2.0 mg/L were classified as “Low Nitrate,” whereas states with average nitrate levels ≥ 2.0 mg/L were classified as “High Nitrate” (Supplemental Exhibit 1) [24].

Although the state-level analysis prohibited us from adjusting for individual-level familial or genetic thyroid cancer risk factors, our analysis did include data adjusting for 2 other factors contributing to both thyroid cancer risk and incident diagnoses. The first factor was obesity, which is associated with increased thyroid cancer risk [4]. Annual, age-specific obesity and overweight data were accessed from the Behavioral Risk Factor Surveillance System [25]. Childhood obesity data came from State of Childhood Obesity [26]. The second factor contributing to differences in diagnosis, as opposed to risk, is screening; which we measure as time-invariant age-specific population state-level endocrinologist access [27].

### Statistical analysis

After reporting the observed incidence rates (cases per 100 000 population) by sex, age group, and nitrate level we conducted a series of statistical analyses to test if, after adjusting for population, obesity/overweight, and endocrinology access, thyroid cancer cases were higher in states with higher nitrate. Our primary model was a generalized estimating equation (GEE) Poisson regression model, with exchangeable correlation matrix.

As an alternative sensitivity check specification, we constructed a generalized least squares random effects (GLS RE) Poisson regression model. Both models estimated standard errors robust to heteroskedasticity and autocorrelation. Both models included a population exposure offset, essentially modeling differences in cases as an implicit rate differential. Both models adjusted for current and 7-year lagged obesity, as well as overweight rates, and the proportion of the age-specific population with access to an endocrinologist. In addition to estimating absolute modeled differences in cases, we estimated each model with a log-transformed dependent variable to quantify the percentage difference in cases between high and low nitrate states.

## Results

### Summary statistics

Table 1 presents the incidence rates of thyroid cancer per 100 000 population, grouped by nitrate exposure (low vs high). Individuals within the early-onset adult age group (40-59) had the highest overall incidence rate of thyroid cancer (21.42 cases per 100 000), followed by those in the age group of 15 to 39 (10.23 cases per 100 000). The 0 to 14 age group had the lowest overall incidence of thyroid cancer (0.31 cases per 100 000). For all age groups, overall and in both males and females, the observed incidence of thyroid cancer was higher in the high nitrate exposure states than the low nitrate exposure states. Supplemental Exhibits 2 to 4 report and visualize the observed, population-adjusted thyroid cancer cases by nitrate group, age, and sex [24].

**Table 1:** Summary statistics—thyroid cancer incidence rate (cases per 100 000)

	Overall	Low nitrate	High nitrate
Age 0 to 14	0.31	0.30	0.32
Age 15 to 39	10.23	9.39	10.87
Age 40 to 59	21.42	19.89	22.61
Age 0 to 14 females	Suppressed	Suppressed	Suppressed
Age 15 to 39 females	17.03	15.70	18.07
Age 40 to 59 females	32.14	29.77	33.98
Age 0 to 14 males	Suppressed	Suppressed	Suppressed
Age 15 to 39 males	3.60	3.23	3.88
Age 40 to 59 males	10.44	9.72	11.01

Table 1 reports the thyroid cancer incidence rate (cases per 100 000 population) overall and by low (< 2 mg/L) and high (≥ 2 mg/L) nitrate state groups. The incidence rate for sex-stratified pediatric populations was suppressed by NPCR (*n* < 16 cases).

### Primary results

**Figure 1:**
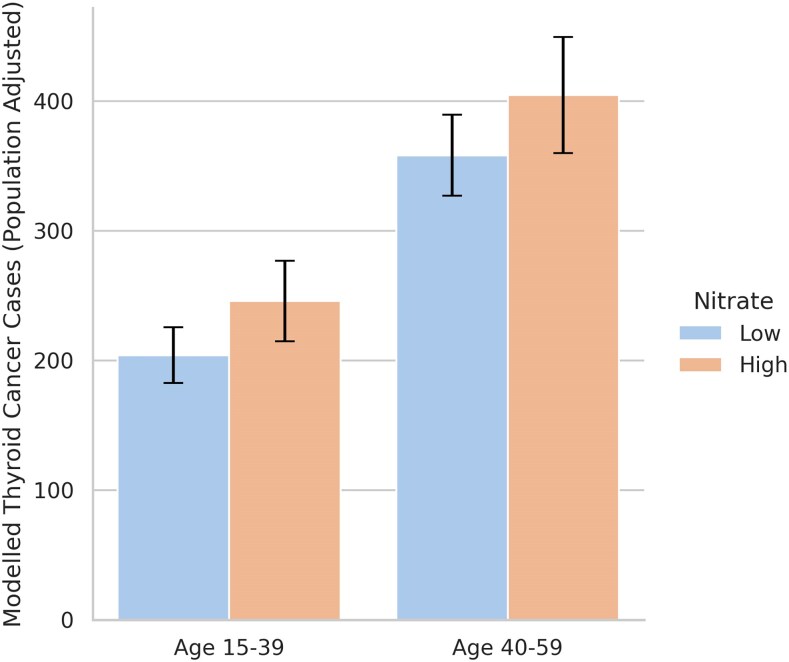
Modeled thyroid cancer cases (adjusted for population) by age group and nitrate level. Figure 1 visualizes the modeled difference in population-adjusted thyroid cancer case counts from generalized estimating equation (GEE) Poisson regression models. Models adjust for state random effects and year fixed effects, as well as current and 7-year lagged state-level rates of obesity and overweight BMI, and measures of endocrinology care access. Error bars represent 95% confidence intervals. High nitrate indicates states with ≥ 2 mg/L average groundwater nitrate based on predicted measures.

**Figure 2:**
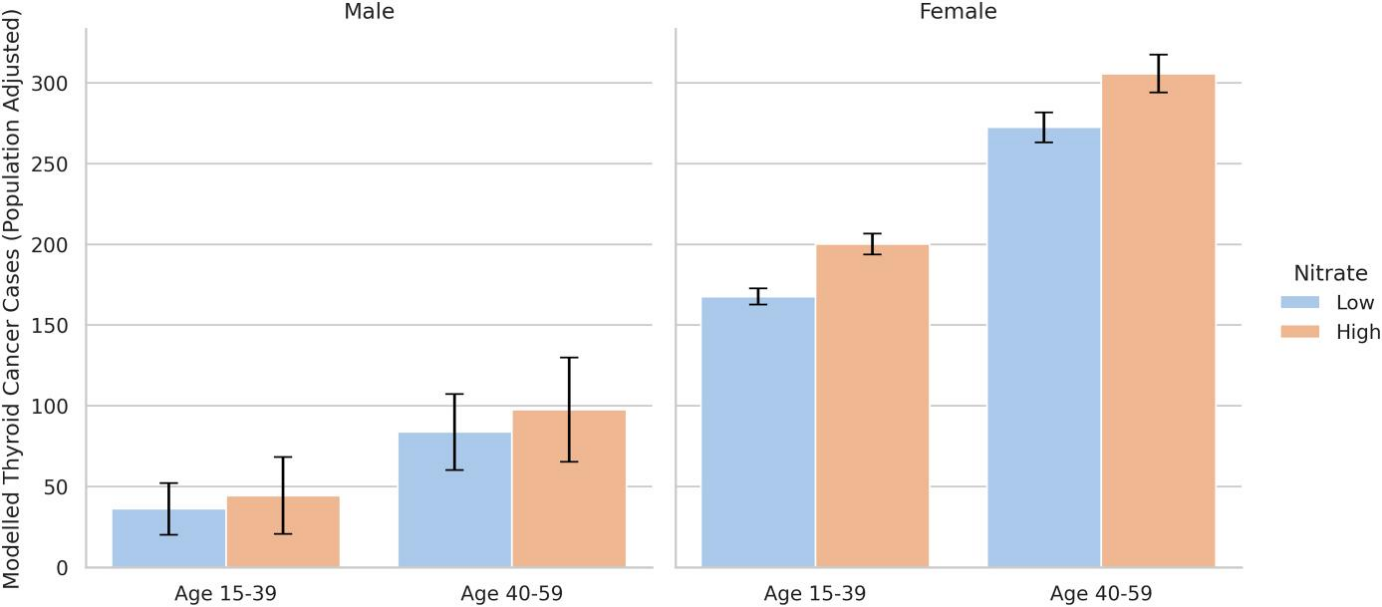
Observed thyroid cancer cases (adjusted for population) by sex, age group, and nitrate level. The figure visualizes the sex-stratified, modeled difference in population-adjusted thyroid cancer case counts from generalized estimating equation (GEE) Poisson regression models. Models adjust for state random effects and year fixed effects, as well as current and 7-year lagged state-level rates of obesity and overweight BMI, and measures of endocrinology care access. Error bars represent 95% confidence intervals. High nitrate indicates states with ≥ 2 mg/L average groundwater nitrate based on predicted measures.

Figures 1 and 2 visualize the population-adjusted, modeled differences in thyroid cancer cases by state nitrate level by age group and sex. Table 2 reports the modeled differences in thyroid cancer cases, between high and low nitrate exposure states. We did not find any differences between high and low nitrate states for pediatric age groups 0 to 14. For those between the ages of 15 to 39, adolescent/young adult (AYA) there were 41.6 (CI: 6.2, 77.1) more cases for those in high nitrate states. This reflects an 18.5% difference. Stratified by sex, in the AYA group females had 32.7 (CI: 6.3, 59.1) more cases and males had 8.2 (CI: 0.1, 16.4) more cases, reflecting a 17.8% and 20.4% difference, respectively.

**Table 2:** Population average GEE Poisson regression—modeled differences in thyroid cancer cases

	Est. [95% CI]	% Difference [95% CI]
Age 0 to 14	0.0 [−0.5, 0.5]	0.5% [−10.4%, 11.5%]
Age 15 to 39	41.6* [6.2,77.1]	18.5% [3.7%, 33.9%]
Age 40 to 59	46.4 [−5.1,97.8]	12.2% [−1.1%, 25.4%]
Age 15 to 39 females	32.7* [6.3,59.1]	17.8% [3.8%, 31.8%]
Age 40 to 59 females	33.2 [−3.3,69.7]	11.5% [−0.9%, 23.9%]
Age 15 to 39 males	8.2* [0.1,16.4]	20.4% [0.4%, 40.4%]
Age 40 to 59 males	13.8 [−1.3,29.0]	15.3% [−1.3%, 31.8%]

Table 2 reports the results of the generalized estimating equation (GEE) Poisson regression models estimating (Est.) the association between high nitrate levels (≥ 2 mg/L) and thyroid cancer cases. All models adjusted for population. All regression models adjusted for current and 7-year lagged state-level rates of obesity and overweight BMI, and measures of endocrinology care access. Standard errors were estimated robust to heteroskedasticity and autocorrelation. Ninety-five percent confidence intervals (CI) reported in brackets. Table 2 also reports the results of a post-GEE model marginal analysis, estimating the relative difference on a 0 to 100% scale. *P < .05, ** *P* < .01, *** *P* < .001.

Conversely, for early-onset adult populations between ages 40 and 59, there were no statistically significant differences between state nitrate groups. However, the 95% confidence interval rules out large negative differences (< −5.1 cases or <1.1%) in the high nitrate states. More simply, with high certainty we can claim that low nitrate states will not have meaningfully larger incidence of thyroid cancer in early-onset age groups. For AYA populations aged 15 to 39 years, GLS RE estimates were consistent for the overall group and in females (but not males). The GLS RE estimates (null) for the pediatric and early-onset age group were consistent with the GEE estimates.

## Discussion

With rising levels of thyroid cancer, it is important to understand how exposure to nitrate contamination in drinking water influences incidence. Our research found that thyroid cancer in adolescent and young adult populations was consistently higher in states with high groundwater nitrate exposure compared to states with low nitrate exposure. This association between nitrate exposure and thyroid cancer incidence were minimal and statistically insignificant in pediatric (<15 years old) and adult (40-59 years old) groups. Even after adjusting for obesity and access measures, our models suggested that the largest association between nitrate exposure and thyroid cancer incidence was found in 15 to 39-year-old females.

Thyroid cancer incidence did not differ by nitrate levels in the <15-year age group, a finding that was unsurprising given the rarity of thyroid cancer in childhood, the limited statistical power to detect small exposure-related differences, and shorter lifespan of potential exposure to nitrates in this youngest group. In contrast, the absence of a significant association in adults aged 40 to 59 may reflect the more heterogeneous etiology of thyroid cancer at older ages, where cumulative medical, genetic, and benign thyroid disease–related factors, as well as greater residential mobility and age-related diagnostic imaging, may dilute variation attributable to nitrate exposure. Yet, readers should know that the confidence intervals (CI) for this age group included substantial positive relative differences (20-40%) between high and low nitrate states and only minimal negative differences (approximately –1%). Therefore, although statistical significance was not found, our conservative estimation strategy cannot rule out potentially meaningful associations between state-level nitrate exposure and thyroid cancer incidence among 40 to 59-year-old adults.

These results were consistent with many preexisting studies, further suggesting a potential association between nitrate and increased thyroid cancer risk [7, 16, 21, 28]. In addition to focusing on sex-stratified, early-onset thyroid cancer incidence, our major contribution to the existing evidence base was analyzing nationwide cancer data for the entire United States, with models adjusting for both observable heterogeneity (ie, obesity, endocrinologists) and unobservable heterogeneity (random effects) at the state level. Although this study was not designed to quantify a causal relationship; our methodology revealed convincing evidence that reducing nitrate exposure could potentially reduce early-onset thyroid cancer incidence.

Beyond our narrow scope evaluating the association between water-based nitrate exposure and early-onset thyroid cancer incidence, other epidemiological research has shown broader implications related to nitrates and adverse population health outcomes. Recent reviews underscore that nitrate is one of several endocrine disruptors [29]. Other disruptors, such as perchlorates and thiocyanates, may also increase risk of thyroid disease [29]. Another disruptor, arsenic, appears to harm health independently but also synergistically with nitrate exposure [30, 31]. Even at levels complying with regulatory requirements, exposure to nitrates are associated with increased risk of adverse outcomes across the life course [20, 21, 32]. Finally, building upon environmental exposure, evidence continues to show that higher dietary nitrate intake is associated with increased thyroid cancer risk, reinforcing the plausibility of nitrate-related thyroid dysregulation across multiple exposure pathways [33, 34]. Within the context of these broader findings, our study reiterates the importance of continued investigation understanding and mitigating the risk of nitrate-related thyroid disease.

To better understand any causal relationships and underlying mechanisms, future research should utilize individual-level data with quasi-experimental designs. Heterogeneity or mediation analyses could further provide the public with critical evidence for policymakers and healthcare providers by identifying which subpopulations may be at greatest risk of increased incidence of early-onset thyroid cancer with elevated exposure to nitrates in water. Additionally, studies investigating the impact of targeted interventions, such as programs or practices aimed at reducing nitrate exposure, could provide valuable insights into how such measures may influence the incidence of thyroid cancer. These future studies can help guide the development of public health strategies and policies aimed at mitigating the adverse effects of nitrates on cancer.

The emerging epidemiology evidence, backed by the well-established multifaceted biological mechanisms linking nitrates to thyroid cancer warrant not just greater research attention to understand the role of nitrate, but greater regulatory attention to mitigate the adverse effects of nitrates in drinking water with elevated nitrate on the development of early-onset thyroid cancer [7, 9, 10, 28, 35]. The current EPA drinking standard of 10 mg/L in water was set in response to methemoglobinemia, with no regards to cancer [15, 36, 37]. Regulatory efforts must include a portfolio of protections for public water systems as well as private well owners [38]. However, to effectively reduce the risk of early-onset thyroid cancer incidence by reducing exposure to groundwater nitrate contamination, policymakers must aim upstream by incentivizing and requiring improved agricultural nitrate reduction practices [39-41].

### Limitations

Our study is not without its limitations. First, the analyses were observational and therefore cannot establish causality. While we identified associations between nitrate levels and thyroid cancer incidence, these results may be subject to residual confounding, reverse causation, or other sources of bias inherent to ecological studies. Thyroid cancer has a multifactorial etiology, with suspected risk factors such as radiation and hormonal influences [6, 42, 43]. These factors, which could potentially be driven by environmental contaminants, likely vary across populations, age groups, and geography [44]. Whether our results are confounded by these other factors requires further investigation. Second, the models used were complex and our conservative inference resulted in wide CI, potentially increasing Type II error. Third, our measure of nitrate exposure relied on a single state-level classification derived from archived county-level groundwater data (1991-2010). Although this represented the best available nationwide data, it may not accurately reflect more dynamic changes in nitrate levels or within-state heterogeneity. Further, the nitrate measures from 1991-2010 do not perfectly overlap with the thyroid cancer incidence data (2003-2022). This incongruence may raise the possibility of exposure misclassification. However, the earlier nitrate data provides a window for capturing exposure relevant to adult-onset thyroid cancer. Moreover, these USGS measurements represent the only comprehensive nationwide dataset overlapping the cancer data.

Because thyroid cancer incidence data were not available at the county level by age and sex, we were unable to evaluate finer geographic or subgroup variation, which could obscure more granular associations with higher nitrate exposures. Additionally, our reliance on state-level, aggregated data introduces the risk of ecological fallacy. Associations identified at the state level may not apply to individuals within those states, and therefore readers should avoid causal interpretations of our study.

## Conclusions

In summary, thyroid cancer was consistently higher in those aged 15 to 39 residing in high ground water nitrate states. Regulation of nitrate contamination in drinking waters at the source may be protective against the development of early-onset thyroid cancer in adolescents and young adults. Future studies utilizing individual level, prospective data could help guide the development of future public health strategies and policies.

## Data Availability

Original data generated and analyzed during this study are included in this published article or in the data repositories listed in Reference [24].
